# The rate of change in declining steroid hormones: a new parameter of healthy aging in men?

**DOI:** 10.18632/oncotarget.11752

**Published:** 2016-08-31

**Authors:** Andreas Walther, Michel Philipp, Niclà Lozza, Ulrike Ehlert

**Affiliations:** ^1^ Clinical Psychology and Psychotherapy, University of Zurich, Zurich, Switzerland; ^2^ University Research Priority Program – Dynamics of Healthy Aging, University of Zurich, Zurich, Switzerland; ^3^ Psychological Methods, Evaluation and Statistics, University of Zurich, Zurich, Switzerland

**Keywords:** aging, sex steroids, decline, biomarker, psychosocial factors, Gerotarget

## Abstract

Research on healthy aging in men has increasingly focused on age-related hormonal changes. Testosterone (T) decline is primarily investigated, while age-related changes in other sex steroids (dehydroepiandrosterone [DHEA], estradiol [E2], progesterone [P]) are mostly neglected. An integrated hormone parameter reflecting aging processes in men has yet to be identified. 271 self-reporting healthy men between 40 and 75 provided both psychometric data and saliva samples for hormone analysis. Correlation analysis between age and sex steroids revealed negative associations for the four sex steroids (T, DHEA, E2, and P). Principal component analysis including ten salivary analytes identified a principal component mainly unifying the variance of the four sex steroid hormones. Subsequent principal component analysis including the four sex steroids extracted the principal component of declining steroid hormones (DSH). Moderation analysis of the association between age and DSH revealed significant moderation effects for psychosocial factors such as depression, chronic stress and perceived general health. In conclusion, these results provide further evidence that sex steroids decline in aging men and that the integrated hormone parameter DSH and its rate of change can be used as biomarkers for healthy aging in men. Furthermore, the negative association of age and DSH is moderated by psychosocial factors.

## INTRODUCTION

Aging, per se, dramatically increases the vulnerability to pathology and disease [1–4]. The current demographic shift towards a significantly older population is leading to an unprecedented rise of disability and dependence in our society. Health care costs are expected to rise enormously [5, 6]. Therefore, one of the most crucial health care goals for the upcoming decades is to prolong health span and delay aging itself [7]. To achieve this goal, new strategies and methods are required to prevent unhealthy aging already in early adulthood. Dysfunctional aging-patterns need to be identified and high sensitivity to maladaptive changes has to be provided, so that signs of declining health may be easily detected and successfully addressed [7, 8].

Over the last 25 years, research on healthy aging in men has increasingly focused on age-related hormonal changes starting around the age of 40. In the aging male, the decline in free testosterone (fT) of 2-3% per year has been linked to age-related clinical conditions such as frailty, erectile dysfunction, or depression [9, 10]. The syndrome of late-onset hypogonadism (LOH) has been defined as serum fT levels below 220 pmol/l and the presence of at least three sexual symptoms such as erectile dysfunction or low sexual desire [9]. The overall prevalence of LOH in the European Male Aging Study, which included 3369 men aged 40 to 79 years, was 2.1%; the number was significantly higher for the age groups 60 to 69 years (3.2%) and 70 to 79 years (5.1%) [9].

Recent studies show similar importance of low levels of dehydroepiandrosterone (DHEA), estradiol (E2), and progesterone (P) for different areas of life (psychological, sexual, cognitive, and physical functioning) within the aging men 11-13]. Still, low testosterone (T) levels are mostly the solely target of therapeutic treatments in relation to LOH while the clinical relevance of low levels of DHEA, E2, or P generally tends to be neglected [14]. Due to the strong interrelation between steroid hormones, the single assessment of one steroid hormone only seems to be insufficient to adequately reflect the hormonal state of an individual. For example, a hormonal imbalance could be expressed by high T levels while the expression of DHEA and P are below a critical range. Therefore, examining only a single hormone may lead to wrong interpretations in some cases [15, 16].

Due to large inter-individual differences in hormone concentrations agreement on norm values for circulating blood or saliva levels is still missing. While literature generally agrees on age-dependent progressive decline of free T, DHEA, and E2 [17–19], conflicting data on age-related free P-decline in men is reported [20, 21]. It has become clear, however, that not only age-related changes in T, but also in DHEA, E2, and potentially also in P, are responsible for a variety of diseases met by aging men such as dementia, frailty, depression, or sexual dysfunction [22–25].

Therefore, the rate of change in declining steroid hormones seems to be a robust parameter of health deterioration. Higher endogenous levels of T, DHEA, E2, and P in older men are related to better mental and physical health and a slower decline of these steroids with age is regarded as health protective [26, 27]. In addition, apparent and self-reported good health have been shown to be associated with attenuated age-related sex steroid decline in men [17, 28]. In line with these findings, a follow up examination after four years of men aged 40 to 79 showed weight gain of more than 10% to be associated with a greater decline in T [29]. To our knowledge, no psychosocial factors have been associated with reduced or increased age-related sex steroid decline in men to date.

The primary aim of the present study was to identify a comprehensive biological indicator of healthy aging in men that provides high sensitivity to identify early onset health deterioration, even before symptoms are perceived. Additionally, this parameter should be robust enough to allow large inter-individual differences, and it should be easy to assess. Additionally, we were interested in examining possible associations between depression, chronic stress, stressful life events and health status with the rate of change in declining steroid hormones.

## RESULTS

Subject demographics and selected laboratory characteristics are summarized in Tables 1 and 2.

**Table 1:** Characteristics of the sample

Total	*N* = 271	% = 100
Age (Mean / SD)	57.1	10.7
Current health condition (N / %):		
Very good	97	35.8
Good	148	54.6
Fair	26	9.6
Bad Very bad	0 0	0.0 0.0
Body mass index (kg/m2) (Mean / SD)	25.4	3.4
Education (N / %):		
tertiary education	106	39.1
post secondary non-tertiary education	57	21.0
Higher secondary school	76	28.0
Lower secondary education Did not finish regular school	32 2	11.1 0.8
Current smoking status (N / %): Non smoker Occasional smoker Smoker	224 25 22	82.7 9.1 8.2
Medication intake (N / %): No Yes Medication type used^a^ (N / %): Antihypertensives Antidepressants Anti-inflammatory or painkiller Others	180 91 40 7 28 49	66.4 33.6 14.8 2.6 10.3 18.1

a Participants could indicate to use more than one medication simultaneously.

**Table 2:** Descriptive statistics of sex steroids (pg/mL) and the principal component of declining steroid hormones (DSH)

	Testosterone (T)	Dehydroepiandrosterone (DHEA)	Estradiol (E2)	Progesterone (P)	Principal component (DSH)
N^a^	268	266	270	264	256
Mean (SD)	67.37 (26.72)	256.25 (224.26)	1.32 (.99)	28.43 (18.79)	−.12 (.94)
Minimum	7.77	8.07	.16	.05	−1.54
Maximum	165.06	1129.51	6.0	104.18	3.98

a Absolute number of analyzed hormonal parameter included for statistical analyses. For extraction of DSH a principal component analysis with T, DHEA, E2 and P was performed. Only participants that provided saliva samples and their samples were not contaminated or values for T, DHEA, E2 or P were not below the detection limit or identified as outliers provided data for the computation of DSH (N = 256).

A principal components analysis for the ten salivary analytes was conducted. T, DHEA, E2, and P loaded on the same principal component. In order to represent the four sex steroids by a single indicator (i.e., through a linear combination of the four sex steroids) with the largest variance, a principal component analysis with one factor was conducted using only the four sex steroids - resulting in the principal component of declining steroid hormones (DSH) (see supplementary material Tables Xa and Xb). Zero-order correlations between age and salivary sex steroids revealed consistently negative associations (T: r = −.345,*p* < .000; DHEA: r = −.385, *p* < .000; E2: r = −.208, *p* < .000; P: r = −.276, *p* < .000). These results are in line with previous findings [13, 17, 18, 20]. Further correlation analysis revealed a negative association between age and DSH (DSH: r = −.416, *p* < .000, see Table Yb in the supplementary material).

Quantile regressions of the single declining steroid hormones and the principal component of DSH as a linear function of age are represented in Figure 3. A mean annual reduction of 1.28% for T, of 3.52% for DHEA, of 1.18% for E2, and of 1.68% for P was calculated. DSH declined per additional year about .042 units, what reflects the mean rate of change in DSH.

**Figure 1:**
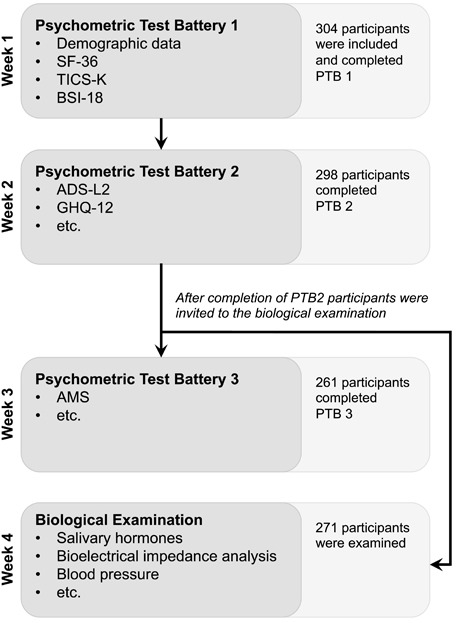
Flow chart of the study procedure and participation

**Figure 2:**
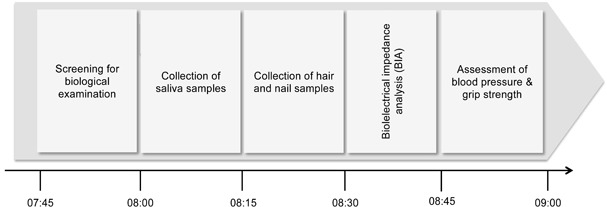
Timeline of the biological examination

**Figure 3:**
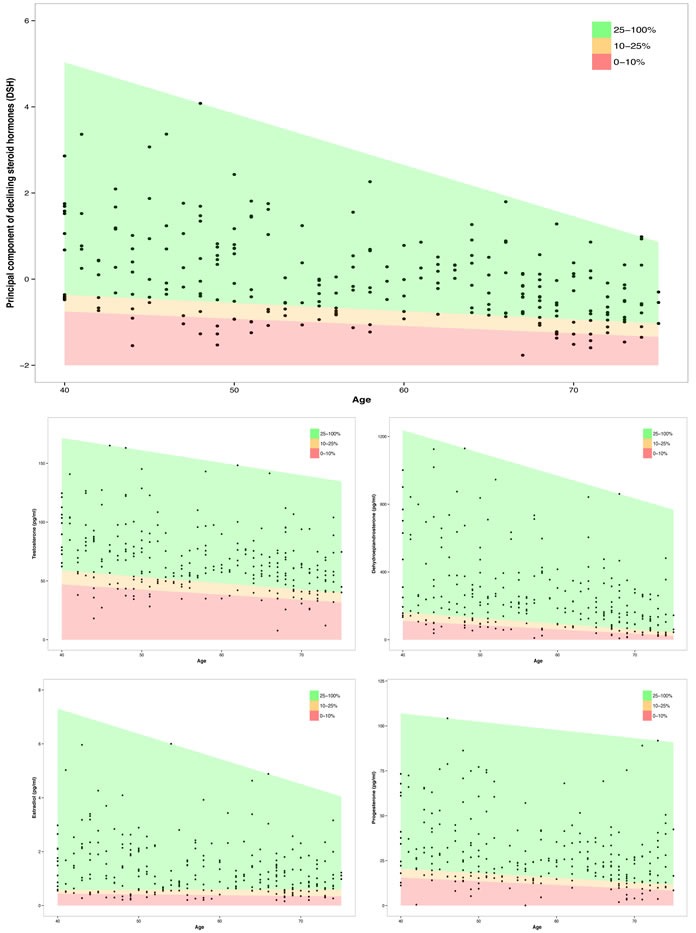
Association between age and the principal component of declining steroid hormones (DSH) and four sex steroids (T, DHEA, E2 and P) for different quantiles (red: lowest 10%; red and orange: lowest 25%; green: upper 75%)

To analyze moderation effects on the relationship between age and DSH, moderation analysis was conducted using two different regression techniques. Moderation analysis by OLS showed a significant association between age and DSH for depressive symptoms (ADS-L2: *β* = −.0017, *p* = .045), chronic stress (TICS: *β* = −.0010, *p* = .019), and perceived general health (GHQ: *β* = .0029, *p* = .035). Further moderation analyses by OLS failed to reach the level of statistical significance.

**Figure 4:**
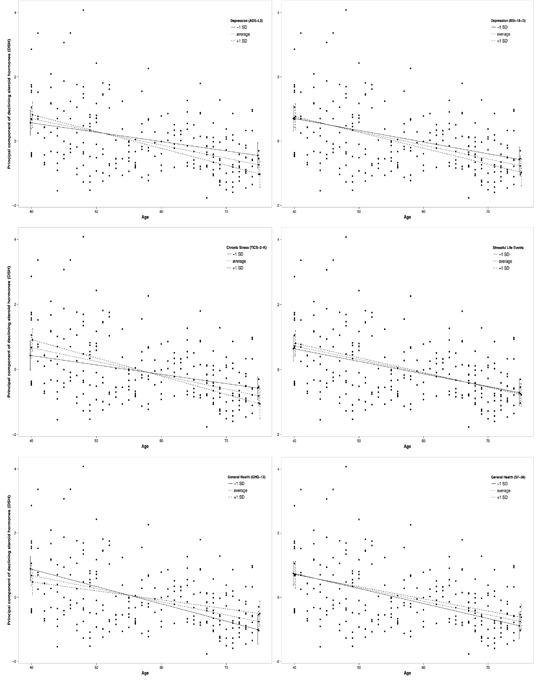
Moderation plots of the associations between age and the principal component of declining steroid hormones (DSH) by depressive symptoms (top left: ADS-L2; top right: BSI-18-D), chronic stress (middle left: TICS-2-K), stressful life events (middle right: SLE), and general health (bottom left: GHQ-12; bottom right: SF-36)

Moderation analysis by using robust regression showed significant associations between age and DSH for depressive symptoms (ADS-L2: *β* = −.0017, *p* = .033; Figure 4), chronic stress (TICS: *β* = −.0010, *p* = .015; Figure 4), and perceived general health (GHQ: *β* = .0029, *p* = .016; Figure 4). Further moderation analyses by robust regression failed to reach the level of statistical significance.

While the mean rate of change in DSH is .042, an increase of one standard deviation in depressive symptoms (ADS-L2) increases the rate of change in DSH to .053. For an increase of one standard deviation in chronic stress (TICS-2-K) the rate of change in DSH mounts from .042 to .056. For an increase of one standard deviation in general health (GHQ-12) the rate of change in DSH decreases from .042 to .028.

Moderation analysis and interaction plots for the association of the four sex steroids and age moderated by psychosocial factors are represented in supplementary material Table Z and Figure A-D.

## DISCUSSION

This manuscript focuses on an integrated hormone parameter, namely the rate of change in declining steroid hormones, as an indicator of healthy aging in men.

The final sample consisted of 271 healthy men between the ages of 40 and 75 years, who provided psychometric data and saliva samples for hormone analysis. As expected, sex steroid hormones (T, DHEA, E2, and P) were strongly inter-correlated. A negative association with age was reported for all four of these sex steroids. An exploratory principal component analysis of all salivary hormones and immune parameters revealed strong evidence for a principal component consisting of the four sex steroids T, DHEA, E2, and P. In order to represent the four sex steroids by a single indicator with largest variance, we computed a principal component of declining steroid hormones (DSH) with T, DHEA, E2, and P. DSH was negatively associated with age. DSH decreased .042 units per additional year in the entire sample, while T or DHEA decreased 1.28% or 3.52% per year, respectively. The annual decline of DSH reflects the rate of change in declining steroid hormones. The rate of change in DSH is moderated by depressive symptoms, chronic stress, and general health - all to a small degree. More depressive symptoms and chronic stress seem to amplify age-related sex steroid decline, whereas better general health seems to buffer against age-related sex steroid decline. Depressive symptoms and chronic stress increase the rate of change in DSH and better general health reduces it.

A continuous age-related decline in different sex steroid hormones in men has been reported consistently [17–20]. In this study, all four sex steroids were negatively associated with age, which agrees with much of the current literature. There is, however, also substantial conflicting literature [21]. Age-related decline of total T, DHEA, E2, or P in blood serum is more difficult to capture than free sex hormone levels because of an age-related increase in sex hormone binding globulin that additionally reduces free sex hormone levels (SHBG). Therefore, levels of free or bioavailable T, DHEA, E2, or P seem to decline more consistently [18, 30, 31], even though there is substantial conflicting literature [32]. Measurement of sex steroids in saliva, as opposed to in blood serum, has the advantages of directly quantifying the hormone in its unbound form, being a low invasive procedure and producing very low costs.

Already in the 1980's, Wang and colleagues or Vittek and colleagues have shown salivary hormone measurements to be equivalent to blood hormone measurements [33, 34]. Gavrilova and Lindau found that the collection of salivary specimens in older men is feasible and they provide relevant information on conditions of aging [32]. The levels of salivary sex steroid hormones in this sample are similar to other samples of males aged 57 to 85 and reflect a typical age-related sex steroid decline, as compared to younger male samples [30, 35].

Considerable interest is paid to T decline and its treatment in men while the decline of other relevant sex steroids is widely neglected [36]. Although widespread benefits have been reported for DHEA supplementation in men and a relevant contribution to sexual health and body composition in men has been shown for E2 11,13,26,37], free DHEA or E2 decline has not been sufficiently addressed [14]. Instead of neglecting this decline and focusing only on T decline, DHEA, E2, and P decline could be used to further strengthen an integrated parameter capturing aging processes in males.

Different methodological and theoretical problems do arise when using only one hormonal parameter as an indicator of declining health in men [9, 10, 38]. A hormonal parameter involving information from different outcome measures is much more robust against bias than, for example, T alone. Furthermore, not the total amount of circulating sex steroids, but the rate of change in DSH, provides a sensitive parameter of the hormonal aging process in groups and, if measured repeatedly, in individuals. As a steeper decline of sex steroids is recognized as health compromising [17, 27], the information provided by this parameter can be used as the basis for future interventions addressing endocrine health in aging males.

Sex steroids are highly inter-correlated (see supplementary material Table Ya). Following others in the field, we conducted a principal component analysis of salivary analytes [39, 40]. Our analysis showed that the four sex steroids (T, DHEA, E2, and P) are loading strongly on the same principal component. Therefore, only these four sex steroids were included to compute the principal component of declining steroid hormones (DSH) reflecting only variance of these four sex steroids in DSH (see supplementary material Table Xb).

The negative association of DSH and age is moderated by psychosocial factors in healthy men. The relationship of depressive symptoms and sex steroids is discussed controversially within research. However, a great body of literature supports the perspective that higher levels of T, DHEA, and E2 in older men are negatively associated with depression [9, 41–44]. For P, studies in post menopausal women indicate that decreased P levels are a risk factor for depression [45]. In men, decreased plasma P levels have been found during a major depressive episode, but the sample size of the study is too small to draw final conclusions [46]. Visual analysis of the interaction plots for two depression measurements (ADS-L2 and BSI-18-D) show consistently higher levels of depressive symptoms associated with a steeper age-related decline of T, DHEA, E2, P, and DSH. The fact that in our study moderation analysis revealed significant effects for the association of age and DSH moderated by ADS-L2 but not by BSI-18-D, might be due to the precision of depression measurement of the two instruments. While ADS-L2 consists of 20 items and reflects a broader spectrum of depression measurement, BSI-18-D consists of only 6 items and is generally considered a brief screening tool.

Chronic stress and stressful life events are considered to attenuate sex steroid secretion [47]. For different sex steroids, a stress reducing effect is discussed for males [48]. In our study, more chronic stress measured with the TICS-2-K revealed an amplifying effect on the negative association between DSH and age. The two-item factor of stressful life events (amount and quality) failed to significantly moderate this association. These results concur with previous work indicating that chronic stress negatively influences healthy aging and contributes to health deterioration reflected by biological markers [49].

A steeper sex steroid decline with age is considered health compromising [27], while better general health decelerates age-related sex steroid decline [17, 28]. Our results further support this finding. The negative association between age and DSH is significantly buffered by better general health measured with the GHQ-12. Visual analysis of the interaction plot shows the same tendency for general health measured with the SF-36, but it fails to become significant in the moderation analysis. By contrast, neither a significant moderation effect nor a visual detectable trend has been observed for AMS. This might be due to the fact that the AMS measures 17 perceived symptoms related to an advanced aging process and androgen dysfunction. In our study, only self-reporting healthy men were included causing perhaps too little variance in the AMS.

**Figure 5:**
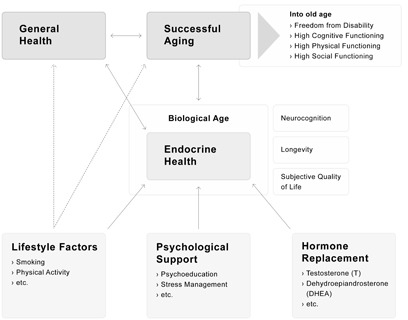
A model of successful aging: Biological aging as main contributor of successful aging Endocrine health, operationalized by the rate of change in DSH, reflects within biological aging a possible target to decelerate biological aging *via* lifestyle factors, psychological support or hormone replacement.

Taken together, the new parameter DSH has been validated by these well-studied constructs in our sample of healthy men. As shown in Figure 5, our results lend further evidence for a model of healthy and successful aging. Life style factors, psychological factors, and general health, as well as hormone replacement, promote endocrine health and, therefore, help to maintain successful aging in men.

There are several limitations that need to be considered when interpreting our results. One major limitation of this study is its cross-sectional design; causal inferences are not allowed. The hormonal differences in relation to age are between-subject effects and they need to be further examined in relation to hormonal changes over time. Therefore, longitudinal data are required to replicate our between-subject results and to further confirm these in a within-subject design. Another limitation, albeit an intentional one, is that the results only apply to men reporting being in a good health state. The generalizability of our results is restricted to self-reporting healthy men between the ages 40 and 75. Group comparisons between self-reporting healthy men and men with a well defined clinical syndrome are needed to confirm the discriminant validity of the new parameter. Our data indicate that a higher rate of change in DSH is related to higher morbidity and to more age-related disorders. A methodological limitation of the study is the single assessment of the salivary parameters. Steroid hormones tend to show variance between consecutive days. Therefore, repeated measurements on different days and subsequent calculation of a mean for each hormonal parameter would provide higher reliability. Additionally, the psychometric data were measured one to three weeks before saliva samples were obtained. This time gap might have resulted in a considerable bias between psychometric and biological outcomes, however measured psychometric constructs reflect traits rather than states.

In conclusion, the annual decline of DSH, namely the rate of change in declining steroid hormones, has been shown to be a promising parameter for monitoring healthy aging in men. In addition to the measurement of the total amount of single sex steroids, which vary greatly from individual to individual, the rate of change in declining steroid hormones provides a more robust picture of a male's endocrine state. Sex steroid reference ranges might help to initially integrate the hormonal situation of a single male into a context, but repeated measurements over a longer time period, and the monitoring of the rate of change in DSH, would provide a more sophisticated picture of endocrine aging processes and of possible health deterioration. As shown in Figure 5, monitoring the rate of change in DSH enables the development of a timely and effective strategy to maintain endocrine health through eventual psychological interventions, life-style interventions or hormone supplementation. Hormonal changes occur usually long before symptoms are perceived and disorders are established. In order to effectively prolong the span of good health in men, this new integrated parameter allows for action before health damage has become an irreversible reality.

## MATERIALS AND METHODS

### Participants

Two hundred seventy-one men between the ages 40 and 75 years (M_age_ = 57.06; SD_age_ = 10.68) provided both psychometric and biological data in the study Men's Health 40+. Subjects were recruited through web pages and flyers. Further inclusion criteria for the study, were good knowledge of German and being healthy (not having any acute medical or psychiatric disorder). The health state was controlled by a health question from the first psychometric test battery “How would you describe your current health condition?” with the response options from “very bad” to “very good”.

Table 1 shows the sample characteristics including age, current health condition, body mass index (BMI), education according to the International Standard Classification of Education [50], smoking status, medication intake and medication type.

The study protocol was approved by the local ethics committee of the University of Zurich. All subjects provided written informed consent.

### Procedure

The participants processed psychometric test batteries 1 to 3 (PTB1-3) independently online for three consecutive weeks. 271 participants attended the biological examination in the fourth week. Examination sessions took place individually or in groups of maximum three participants and lasted approximately one hour and fifteen minutes. Figure 1 shows a flow chart of the study procedure and participation.

The day of the biological examination the participants were fasting overnight. Upon arrival, the participants were instructed about the following procedures and a short activity screening was performed. Standardized saliva sampling started at 8:00am to control for diurnal variation of hormone secretion. Subsequently, body measures, such as height, weight, body cell and fat mass, or cardiovascular parameters were measured according to the standardized test procedure between 8:30am and 9:15am (see Figure 2).

### Psychometric Tests

#### Depressive symptoms

Aging has been shown to be accompanied by an increase in mild to moderate depressive symptomatology [1]. Furthermore, not only steroid hormones [51–53], but also markers of inflammation have been shown to be reciprocally related to depressive symptoms [54]. Therefore, we used the depression scale *Allgemeine Depressionsskala-Langform 2* (ADS-L2) to assess participants' depressive symptomatology [55]. As the ADS-L2 is based on the CES-D scale, which is a self-report depression scale for research in the general population [56], it was well suited for this non-clinical sample. We also used the depression subscale of the Brief Symptom Inventory-18 (BSI-18-D) to obtain an additional score for depression [57].

### Chronic stress and stressful life events

As chronic stress reflects a major health concern and contributes to the etiology of several age-related disorders such as cardiovascular disease or Alzheimer's disease [58–60], it serves as well studied risk factor for dysfunctional aging-patterns. Chronic stress has been shown to be related to decreased sex steroid secretion or elevated cortisol levels as well as elevated inflammation [61, 62]. Chronic stress was assessed through the multidimensional *Trier Inventar zur Erfassung von chronischem Stress - Version 2 - Kurzversion* (TICS-2-K), which asks participants on the frequency of stressful situations in the last 3 months [63]. Stressful life events (SLE) are discussed to play a specific role in precipitating both mental and somatic disorders [64]. Since the amount of SLE increases as a function of age, it is assumed to contribute to the pathophysiology of non-healthy aging. As people differ in frequency and intensity of experienced stressful life events, we assessed the amount of experienced stressful life events and discriminated 8 categories with two self-rate items. Participants were asked “How many stressful life events have you experienced?” with response options from 0 to 6 and 7 or more. Subsequently, a list of 8 dimensions of stressful life events was presented, where more response options were possible to choose (e.g. death of a loved one, serious illness, poverty in childhood, or physical abuse in childhood). Following Sali and colleagues the amount and the weighted quality of stressful life events were computed to a total score (SLE-S) [65].

### General health

To assess the participants' general health state, 3 different measures (GHQ-12, SF-36 and AMS) were used. The General Health Questionnaire-12 (GHQ-12) measures general psychological health and was primarily conceptualized to be used in the general population [66–68]. The Short Form-36 (SF36) is a multidimensional self-report questionnaire measuring a total score of general health [69]. The Aging Males' Symptoms scale (AMS) assesses age-related symptoms such as excessive sweating, irritability, or less morning erections [70].

### Endocrine parameters

Saliva samples were obtained in a standardized procedure between 8:00am and 8:15am. Participants were asked to fill subsequently 3 salicaps of 2ml capacity (SaliCaps, IBL International GmbH, Hamburg, Germany) with saliva. Out of each sample 10 parameters were analyzed (testosterone (T), dehydroepiandrosterone (DHEA), estradiol (E2), progesterone (P), melationin (M), cortisol (C), alpha-amylase (AA), C-reactive protein (CRP), interleukin-6 (IL-6), and immune globulin A (IgA); for descriptive statistics of sex steroids see Table 2).

All saliva samples were stored at −20°C until required for biochemical analysis. T, E2, P, C, and AA were analyzed in the biochemical laboratory of the Department of Psychology of the University of Zurich, while DHEA, M, IL-6, CRP, and IgA were analyzed in the biochemical laboratory of the Technical University of Dresden.

### Potential covariates and confounders

Covariates included were fat mass and muscle mass measured with the bioelectrical impedance analysis (BIACORPUS RX 4000; BodyComp V 8.5) [11, 29, 71], blood pressure and pulse measured with a blood pressure monitor (Medisana MTX USB 51083) [72], smoking status, alcohol consumption [29, 73], medication intake [74, 75], drug consumption [76], and amount of potency medicine or related drug consumption [77]. Also included as covariates were having had a cold or other disease during the last two weeks [78, 79], having had an injury or bleeding in the mouth in the last days [80], having been physically active within the last 24 hours [73, 81]. To address participants' delay the exact starting time of saliva collection was used as a covariate [82]. Waking time of the day of the biological examination [83], the general sleep quality (PSQI) [16, 84], education [85], income [85], and neuroticism (BFI-K) [86], as well as time being in a relationship [87], and amount of children [88] were further included as covariates.

### Statistical analysis

First, outliers were identified and removed for the salivary parameters. Values that were greater or less than three standard deviations around the mean were removed [89]. For T 1.1% (*n* = 3), for DHEA 1.8% (*n* = 5), for E2 0.4% (*n* = 1), and for P 2.6% (*n* = 7) of values were removed.

In order to reduce dimensionality of the ten outcome variables, we subsequently calculated a principal component analysis with all salivary parameters using varimax rotation and four factors [90]. According to the factor loadings, the four sex steroid hormones (T, DHEA, E2, and P) mainly loaded on the first principal component. Therefore, a second principal component analysis was conducted only with the four sex steroids computing one principal component - principal component of declining steroid hormones (DSH). DSH with 256 observations included was used for further analysis. Subsequently, we computed zero-order correlations between age and salivary sex steroids and DSH (Spearman's rho, one-tailed).

To assess the rate of change in declining steroid hormones for different risk groups of the population, quantile regressions were performed on DSH as well as the sex steroid hormones T, DHEA, E2, and P as a linear function of age (see Figure 3). Quantile regressions were computed for the 10%- and the 25%-quantile of the sample. To illustrate the risk groups of low levels of sex steroids, the areas between the regression lines were colored red (0 - 10%), orange (10 - 25%), and green (25 - 100%).

In a next step, we conducted separate moderation analyses for the relation between age and DSH moderated by depressive symptoms, chronic stress, stressful life events, and general health. Additionally, the potential covariates and confounders introduced before were included for the analyses. A comprehensive residual analysis was conducted for each model, in which we identified three relatively influential data points. Therefore, two solutions were calculated. The first solution was performed through ordinary least squares (OLS) regressions. The second solution was performed using robust regression, which put lower weights on extreme data points to reduce their influence on parameter and standard error estimates [91].

All analyses were conducted using R version 3.2.2. [92]. Quantile regressions were performed with the R-Package “quantreg” and robust regression with the R-Package “robustbase” using the settings suggested by Koller and Stahel [93]. Level of significance was chosen at α = 0.05.

## SUPPLEMENTARY MATERIALS FIGURES AND TABLES


