# Supplementary material for: The rate of change in declining steroid hormones: a new parameter of healthy aging in men?
Source: Oncotarget. 2016 Aug 31;7(38):60844–57. doi: 10.18632/oncotarget.11752 (PMC5308620; doi:10.18632/oncotarget.11752)
Supplement: Supplementary file 1 [file oncotarget-07-60844-s001.pdf]

The rate of change in declining steroid hormones: a new parameter of healthy aging in men?

Supplementary Material

Table Xa
Principal component analysis with 10 salivary analytes.

Rotated Component Matrix [a]

	Component			
	1	2	3	4
Testosterone	.770			
Dehydroepiandrosterone	.582			
Estradiol	.727			
Progesterone	.748			
Cortisol			.622	
Alpha-Amylase				.826
Melatonin			.514	.566
Interleukin-6		.823		
C-Reactive Protein			.650	
Immunglobulin A		.792		

Extraction method: Principal Component Analysis.
Rotation method: Varimax with Kaiser-Normalization.
Lower values than .5 were suppressed.

a. The rotation converged in 6 iterations.

Table Xb
Principal component analysis with four sex steroids extracting DSH.

Component Matrix [a]

	Component
	1
Testosterone	.777
Dehydroepiandrosterone	.705
Estradiol	.689
Progesterone	.758

Extraction method: Principal Component Analysis.

a. 1 Component extracted.

Table Ya

Zero-order correlations of sex steroids.

	1.	2.	3.
1. Testosterone			
2. Dehydroepiandrosterone	.466**		
3. Estradiol	.360**	.262**	
4. Progesterone	.419**	.353**	.432**

Pearson correlations; ***p* < .01

Table Yb

Zero-order correlations of sex steroids and DSH with age.

	Age
1. Testosterone	–.345**
2. Dehydroepiandrosterone	–.385**
3. Estradiol	–.208**
4. Progesterone	–.276**
5. Principal component of declining steroid hormones (DSH)	–.405**

Pearson correlations; ***p* < .01

Table Z

Moderation analyses (OLS/robust) for the association of the four sex steroids and age moderated by psychosocial factors

Testosterone (T)

	Ordinary least squares regressions (OLS)				Robust regressions			
	Estimate	Std. Error	t value	Pr(>|t|)	Estimate	Std. Error	t value	Pr(>|t|)
Age:ADS-L2	-0.0183	0.0222	-0.8255	0.4099	-0.0227	0.0201	-1.1283	0.2603
Age:BSI-18-D	0.0069	0.0579	0.1191	0.9053	-0.0020	0.0548	-0.0362	0.9711
Age:TICS-2-K	-0.0123	0.0114	-1.0744	0.2837	-0.0111	0.0108	-1.0284	0.3048
Age:SLE-S	-0.0024	0.0053	-0.4583	0.6471	-0.0013	0.0050	-0.2486	0.8039
Age:GHQ-12	0.0264	0.0366	0.7195	0.4725	0.0274	0.0333	0.8233	0.4112
Age:SF36	-0.0005	0.0141	-0.0384	0.9694	0.0066	0.0132	0.5000	0.6175
Age:AMS	0.0121	0.0241	0.5005	0.6172	0.0289	0.0217	1.3351	0.1832

Dehydroepiandrosterone (DHEA)

	OLS Estimate	OLS Std. Error	OLS t value	OLS Pr(>|t|)	Robust Estimate	Robust Std. Error	Robust t value	Robust Pr(>|t|)
Age:ADS-L2	-0.5277	0.1770	-2.9807	**0.0032****	-0.3802	0.1395	-2.7258	**0.0069****
Age:BSI-18-D	-0.6765	0.4730	-1.4303	0.1539	-0.7977	0.3625	-2.2004	**0.0287***
Age:TICS-2-K	-0.0323	0.0935	-0.3454	0.7301	-0.0358	0.0730	-0.4901	0.6245
Age:SLE-S	0.0265	0.0435	0.6084	0.5435	0.0092	0.0337	0.2723	0.7856
Age:GHQ-12	0.5868	0.2965	1.9788	**0.0490***	0.4225	0.2282	1.8518	0.0653^{t}
Age:SF36	0.1510	0.1150	1.3134	0.1903	0.1970	0.0887	2.2209	**0.0273***
Age:AMS	-0.3358	0.1852	-1.8134	0.0712^{t}	-0.2087	0.1521	-1.3723	0.1714

Estradiol (E2)

	OLS Estimate	OLS Std. Error	OLS t value	OLS Pr(>|t|)	Robust Estimate	Robust Std. Error	Robust t value	Robust Pr(>|t|)
Age:ADS-L2	-0.0014	0.0009	-1.6327	0.1038	-0.0011	0.0007	-1.5718	0.1173
Age:BSI-18-D	0.0007	0.0022	0.3275	0.7436	-0.0004	0.0018	-0.1968	0.8441
Age:TICS-2-K	-0.0005	0.0004	-1.1573	0.2483	-0.0003	0.0004	-0.8342	0.4050
Age:SLE-S	-0.0004	0.0002	-1.9508	0.0522^{t}	-0.0004	0.0002	-2.3813	**0.0180***
Age:GHQ-12	0.0012	0.0014	0.8418	0.4007	0.0008	0.0011	0.6824	0.4956
Age:SF36	-0.0001	0.0005	-0.2685	0.7885	-0.0001	0.0004	-0.1897	0.8497
Age:AMS	0.0016	0.0009	1.7898	0.0749^{t}	0.0014	0.0007	2.0469	**0.0419***

Progesterone (P)

	OLS Estimate	OLS Std. Error	OLS t value	OLS Pr(>|t|)	Robust Estimate	Robust Std. Error	Robust t value	Robust Pr(>|t|)
Age:ADS-L2	-0.0186	0.0171	-1.0831	0.2798	-0.0161	0.0147	-1.0976	0.2735
Age:BSI-18-D	-0.0772	0.0443	-1.7421	0.0828^{t}	-0.0590	0.0387	-1.5268	0.1281
Age:TICS-2-K	-0.0211	0.0082	-2.5727	**0.0107***	-0.0169	0.0072	-2.3610	**0.0190***
Age:SLE-S	-0.0050	0.0039	-1.2932	0.1972	-0.0037	0.0033	-1.0944	0.2749
Age:GHQ-12	0.0631	0.0275	2.2986	**0.0224***	0.0458	0.0237	1.9333	0.0544^{t}
Age:SF36	0.0072	0.0104	0.6902	0.4907	0.0091	0.0090	1.0030	0.3169
Age:AMS	0.0034	0.0185	0.1834	0.8546	0.0151	0.0155	0.9732	0.3315

* significant moderation effect on level of significance .05
** significant moderation effect on level of significance .01
^{t} trend – below the level of significance .1

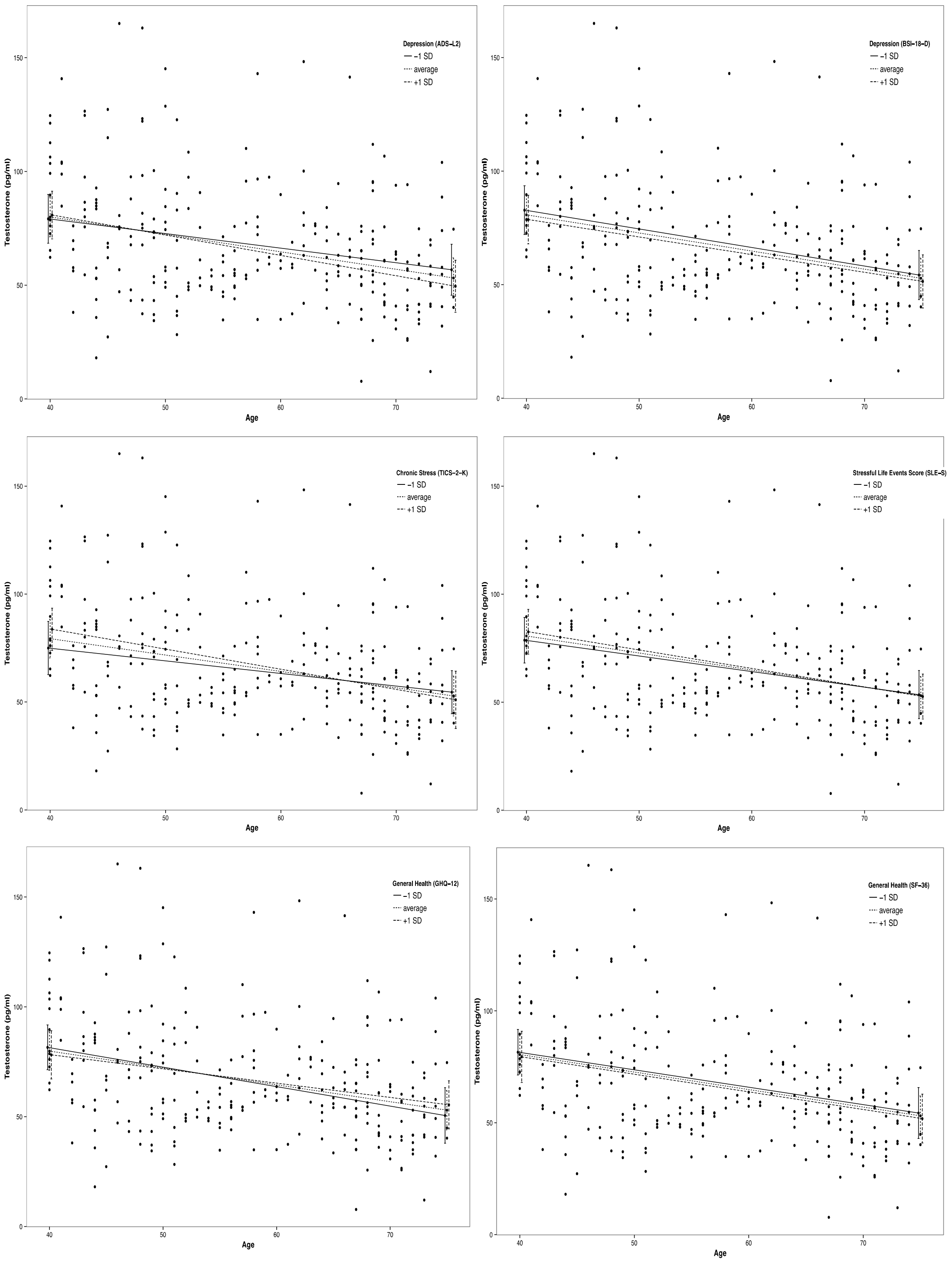

Fig. A. Moderation plots of the associations between age and testosterone (T) by depressive symptoms (top left: ADS-L2; top right: BSI-18-D), chronic stress (middle left: TICS-2-K), stressful life events (middle right: SLE), and general health (bottom left: GHQ-12; bottom right: SF-36).

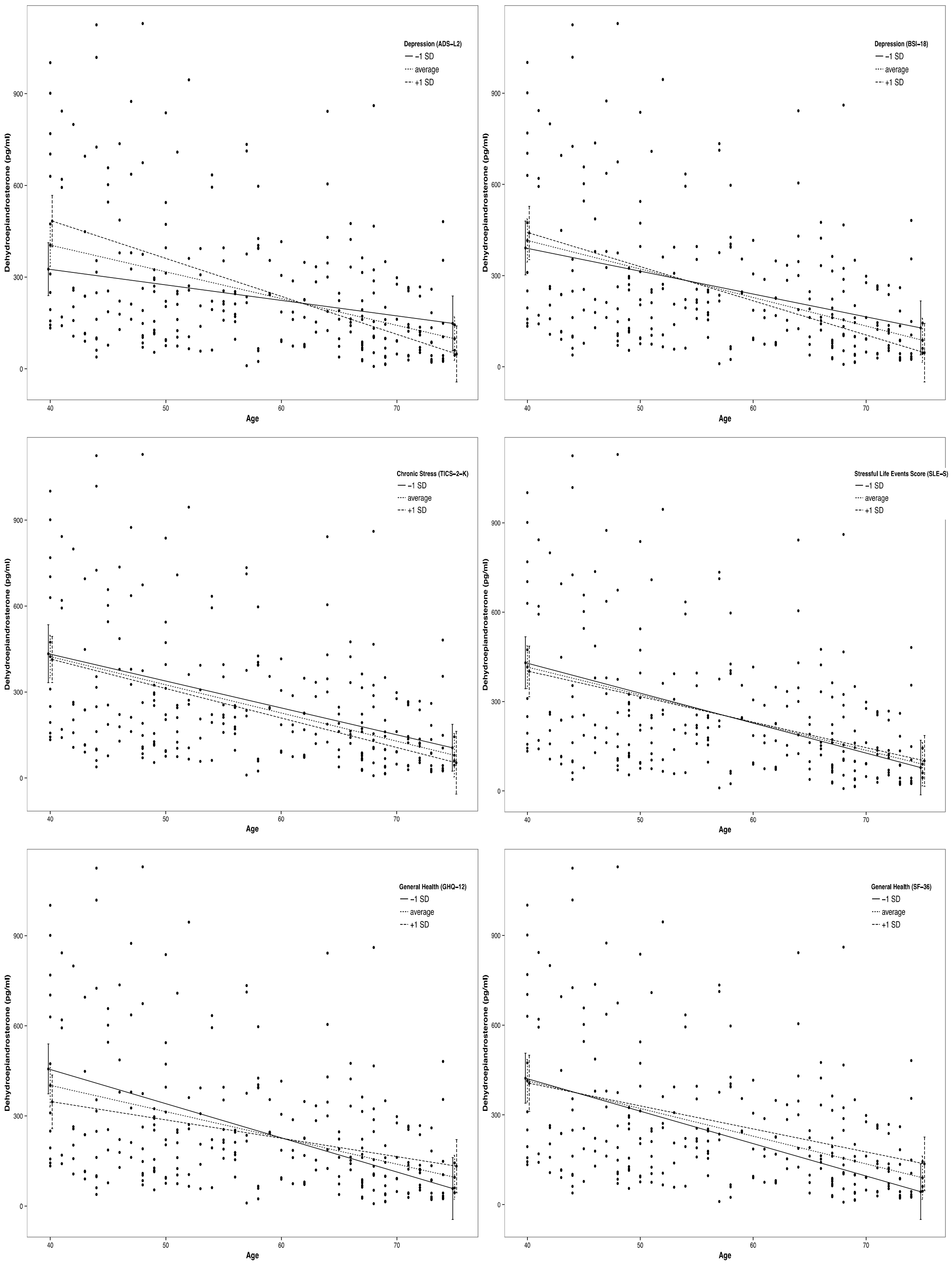

Fig. B. Moderation plots of the associations between age and dehydroepiandrosterone (DHEA) by depressive symptoms (top left: ADS-L2; top right: BSI-18-D), chronic stress (middle left: TICS-2-K), stressful life events (middle right: SLE), and general health (bottom left: GHQ-12; bottom right: SF-36).

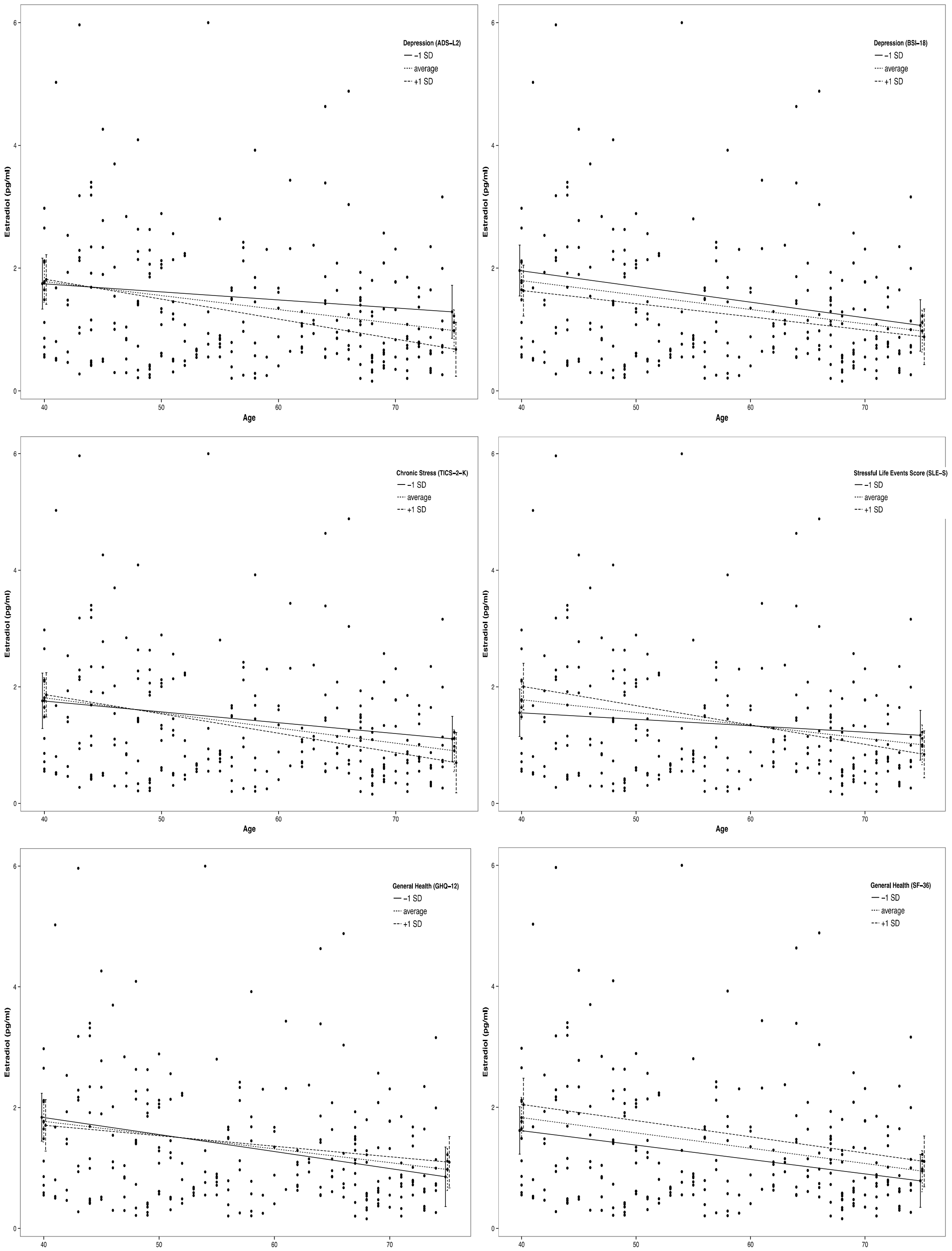

Fig. C. Moderation plots of the associations between age and estradiol (E2) by depressive symptoms (top left: ADS-L2; top right: BSI-18-D), chronic stress (middle left: TICS-2-K), stressful life events (middle right: SLE), and general health (bottom left: GHQ-12; bottom right: SF-36).

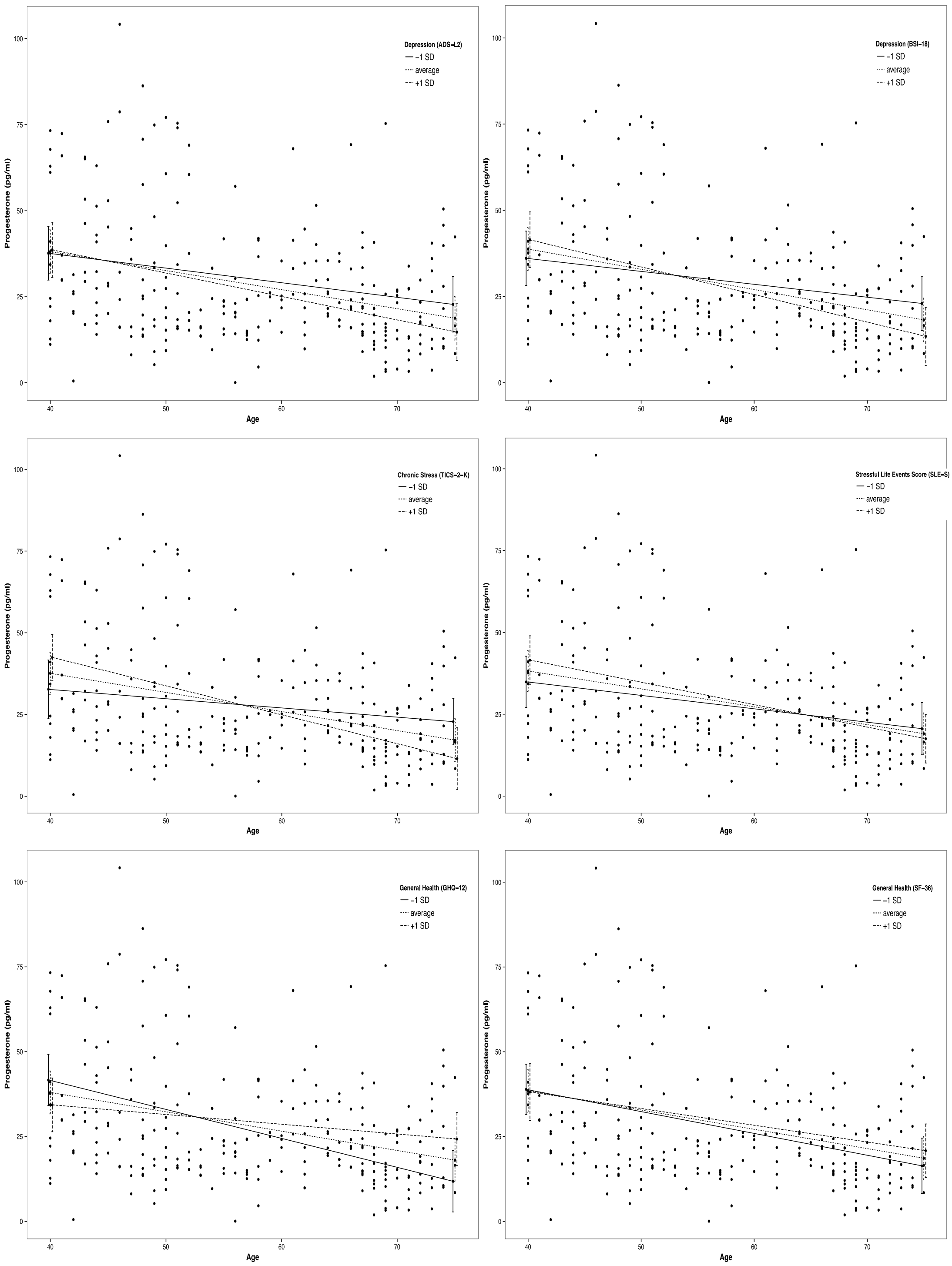

Fig. D. Moderation plots of the associations between age and progesterone (P) by depressive symptoms (top left: ADS-L2; top right: BSI-18-D), chronic stress (middle left: TICS-2-K), stressful life events (middle right: SLE), and general health (bottom left: GHQ-12; bottom right: SF-36).
